# Metabolomics analysis and mRNA/miRNA profiling reveal potential cardiac regulatory mechanisms in Yili racehorses under different training regimens

**DOI:** 10.1371/journal.pone.0322468

**Published:** 2025-07-14

**Authors:** Tongliang Wang, Jun Meng, Xuan Peng, Jinlong Huang, Yunjiang Huang, Xinxin Yuan, Xueyan Li, Xixi Yang, Xiaokang Chang, Yaqi Zeng, Xinkui Yao

**Affiliations:** 1 College of Animal Science, Xinjiang Agricultural University, Urumqi, Xinjinag, China; 2 Xinjiang Key Laboratory of Horse Breeding and Exercise Physiology, Urumqi, Xinjinag, China; 3 Xinjiang Agricultural University Horse Industry Research Institute, Urumqi, Xinjinag, China; Victor Chang Cardiac Research Institute, AUSTRALIA

## Abstract

Yili horses, a versatile breed from Xinjiang, China, are renowned for their racing abilities. However, studies on the links between cardiac morphology, function, and metabolic profiles with performance are limited. This study combined echocardiographic, transcriptomic, and metabolomic analyses to explore these relationships in high-level, average, and untrained Yili horses. Echocardiographic assessments revealed increased left ventricular mass in trained horses, with significant differences in intraventricular septal thickness and left ventricular end-diastolic diameter. RNA sequencing identified 534 differentially expressed genes, 366 differentially expressed miRNAs, highlighting pathways in glycine, serine, and threonine metabolism, oxygen transport (e.g., ALAS2), and ATP generation. Metabolomic analysis revealed variations in acylcarnitine and triglycerides, suggesting training-induced cardiac remodeling regulated by miRNAs. This integrated approach provides new insights into the molecular and metabolic factors influencing performance, offering a foundation for optimized training strategies for Yili horses.

## 1 Introduction

The Yili horse, a breed independently developed in the Xinjiang Uygur Autonomous Region of China, represents a compelling case study in planned breeding. Originating around the turn of the 20th century, the Yili emerged from a deliberate crossbreeding program utilizing native Kazakh horses and imported Russian breeds, including the Don and Orlov Trotter. This strategic introduction of exogenous genetic material aimed to enhance the local equine population with desirable traits from the imported stock.

The breed’s hardiness and adaptability are particularly noteworthy, enabling it to thrive in the diverse terrain and challenging climatic conditions of its native region. Although further research is needed to fully elucidate the genetic diversity and long-term sustainability of the Yili horse population, its historical development and contemporary performance underscore its significance as a valuable equine resource in northwestern China [1].

The Yili horse performance test is a popular equestrian event in Xinjiang, China, and the number of Yili horses registered with the Xinjiang Horse Industry Association has gradually increased to 15,000 (https://horse.xjau.edu.cn/). Yili horses are robust, well-proportioned, and fast with excellent endurance. They are China’s first sport horse with independent intellectual property rights, and their performance has been continuously improved over the years [2].

The heart plays a vital role in delivering oxygen and essential nutrients throughout the body. Echocardiography, a non-invasive technique, is used to examine the heart’s structure and function, especially in sport horses. While research shows that exercise affects heart structure and function in horses, factors like breed, age, and gender also play a role [3], making it important to conduct in-depth studies of specific breeds like the Yili horse. In horses, there is a correlation between intraventricular septal thickness (IVSd) and exercise capacity, and as a horse ages, changes in IVSd can affect cardiac function, which in turn can affect exercise capacity [4]. Exercise also has a significant effect on the LVIDd dimension of the horse’s heart; the LVIDd of horses that compete in races regularly is larger than that in horses that do not compete, and their performance is better. This shows that the intensity and frequency of exercise have a direct impact on heart size, and that horses that regularly engage in high-intensity exercise have larger hearts, higher performance levels, and greater endurance [5]. Relevant studies have shown that with age, there are significant changes in heart weight, heart dimensions, and heart valves [6].

MicroRNAs (miRNAs) are small non-coding RNAs that regulate the expression of numerous genes by binding to mRNA transcripts. They play a critical role in development, cell differentiation, and various diseases. MicroRNAs are not only expressed in tissues but can also be released into the bloodstream. Circulating miRNAs are stable in the bloodstream, but their concentrations change in response to physiological or pathological stress [7]. Circulating miRNAs are strongly associated with exercise and, as convenient biomarkers, they have potential for monitoring and predicting athletic performance, evaluating training outcomes, and assessing exercise-induced injuries and fatigue in horses.

Metabolomics studies have shown that training affects the levels of certain metabolites in horses, which can enhance their performance. For example, comparison of different horse breeds revealed that horses bred for athletic performance exhibit a greater capacity for energy storage and utilization [8]. Lipids are a crucial energy source for horses, especially during endurance activities, and they provide more than twice the energy per gram compared to carbohydrates. Increased dietary fat leads to adaptations in muscle metabolism, allowing for greater utilization of fat as an energy source during exercise. When horses exercise, their bodies break down stored triglycerides into fatty acids. These fatty acids are transported into muscle cells where they contribute to the production of ATP through beta-oxidation [9].

This study focused on understanding how heart structure and function as determined by echocardiography may be correlated with enhanced performance levels in Yili racehorses, and how the differential expression of specific miRNAs can influence metabolites associated with exercise-mediated cardiac remodeling and strengthening of the cardiovascular system. By using an integrated bioinformatics approach, this investigation aims to provide valuable insights into how training can be designed to positively affect heart health and performance in Yili horses.

## 2 Materials and methods

### 2.1 Ethics statement

This study was reviewed and approved by the Animal Welfare and Ethics Committee of Xinjiang Agricultural University (Approval No. 2023037).

### 2.2 Experimental animals and grouping

The study involved 26 Yili horses aged 18 months with similar birth dates (each group consisted of an equal number of male and female horses), minimal differences in body structure, and feeding, and management conditions consistent with accepted husbandry standards (S1 Table). All animals were examined for cardiac and respiratory health and genetic disease. Horses were randomly assigned to a control group without training (BG, n = 6) or to the performance training group (n = 20), which received daily exercise (trot at 50–60% HR_max_, gallop at 70% HR_max_, and slow walk), riding and track training. The heart rate of the horses and exercise intensity were monitored using heart rate monitors and heart rate straps (Supplementary Materials). The six control animals were not trained and were allowed to move freely around the paddock. At the end of the training period, the horses’ performance was tested by running them in a 1000 m race. After a 6-month training cycle, echocardiographic measurements were taken at rest, and horses with larger LVIDd and LVIDs were assigned to the EXC group, while those with smaller measurements were assigned to the CON group. The performance times were 75.68 ± 1.36 s for the EXC group and 78.32 ± 1.97 s for the CON group (S2 Table).

### 2.3 Echocardiography of heart structure and functional parameters

Echocardiograms were obtained from horses in a resting state using a color Doppler ultrasound system (Mindray M6, Shenzhen, China), and analyzed to determine structural and functional parameters of the heart. For the measurements, the horses were brought to a restraint stand, where they were secured and calmed. Body height was measured with a measuring stick, and body weight was determined on a scale. The echocardiographic examination area in the right parasternal region was cleaned and echocardiograms were obtained with a 2.5 MHz probe. The horses’ heart rates ranged from 32 to 45 bpm. The maximum imaging depth was set to 30 cm, with a maximum angle of 110°. A single operator performed three separate imaging sessions, collecting static and dynamic images of the heart at end-diastole and end-systole. These included B-mode long-axis views of the right parasternal area, B-mode images of the right parasternal left ventricular outflow tract, and B/M-mode short-axis images from the right parasternal area. Twenty-two parameters were measured including RVDd, IVSd, LVIDd, LVFWd, RVDs, IVSs, LVIDs, LVFWs, LVminor, MVD, LADd, LADs, AODd, PAd, AODs, PAs, EDV, ESV, EF%, SV, FS%, and LVM. An average value was calculated from three readings.

### 2.4 Transcriptome analysis

#### 2.4.1 Blood sampling.

As approved by the Animal Welfare and Ethics Committee of Xinjiang Agricultural University, all blood samples were collected via jugular vein puncture after alcohol disinfection and transferred into K2-EDTA tubes for hematological testing. A total of 15 mL of blood was collected from the jugular vein of fasting animals, from which 5 mL was mixed with TRIzol reagent at a 1:3 ratio for RNA extraction, 5 mL was used for metabolomics analysis, and the remaining 5 mL was stored at -80 °C for subsequent processing and RT-qPCR transcriptomics analysis.

#### 2.4.2 RNA extraction from whole blood.

Total RNA was extracted with TRIzol at a ratio of 1:3 (blood:TRIzol). The concentration was determined using a Qubit fluorometer and quality was assessed with a Qsep-400 high-throughput Biofragment Analyzer (BiOptic, Taiwan, PRC). Samples with an RNA integrity number (RIN) >7 were selected for subsequent testing.

#### 2.4.3 mRNA library construction and sequencing.

Total RNA was isolated and purified following the manufacturer’s procedure. The RNA amount and purity of each sample was evaluated using a NanoDrop ND-1000 (NanoDrop, Wilmington, DE, USA). The RNA fragment integrity was evaluated through Bioanalyzer 2100 (Agilent, CA, USA). The obtained total RNA was denatured at 70°C for 2 min with DNA 3’ Adapters, and then mixed with T4 RNA Ligase 2, truncated K227Q (NEB, M0351L, USA) at room temperature. The 3’ adapter ligation reaction was performed at 16°C more than 8 hours. The rest of 3’Adapters were removed by RTP at 37°C for 30 min. The 5’ adapters and the products from last step were ligated with T4 RNA Ligase 1 (NEB, M0204L, USA) at 37°C for 60 min, followed by a reverse transcription reaction performed using SuperScript II reverse transcriptase (Thermo, 18064014, USA) at 50°C for 60 min, and then at 80 °C for 10 min. The cDNA products were amplified using the Phusion® high-fidelity DNA polymerase (NEB, M0530L, USA). Specifically, the denaturation was performed at 98°C for 30 s, followed by annealing at 60°C for 30 s, and extension at 72°C for 15 s over 10–16 cycles. The final 5 min extension was then done at 72°C.The PCR products were purified and separated by PAGE. The sequencing strategy was single-end 50 bp for Illumina Hiseq 2500 following the vendor’s recommended protocol.

#### 2.4.4 Identification of mRNA transcripts.

The raw sequencing data in FASTQ format was filtered, sequencing error rates were identified, and GC content distribution was determined to obtain clean data [10]. Clean reads were aligned to the equine reference genome (GCF_002863925.1_EquCab3.0_genomic.fna) using HISAT2 [11] and visualized with IGV [12] software. The reads were spliced into transcripts using StringTie [13].Transcript or gene expression levels were quantified using featureCounts and fragments per kilobase of transcript per million fragments mapped (FPKMs) [14] were computed. Differential expression analysis between sample groups was performed using DESeq2 [15,16] and the Benjamini-Hochberg method was applied to adjust the *p* values and reduce the false discovery rate (FDR) for multiple hypothesis testing. Differentially expressed genes (DEGs) were identified based on the criteria |log2Fold Change| ≥ 1 and *p* < 0.05, After differential expression analysis, the Benjamini-Hochberg method was applied to adjust the *p-*values for multiple hypothesis testing and to obtain the false discovery rate (FDR). The criteria for identifying differentially expressed genes were |log2Fold Change|≥ 1.5 and FDR < 0.05. Gene ontology (GO) enrichment analysis for DE transcripts was performed using R (version 3.5.1) with clusterProfiler (version 4.6.0) and GO.db (version 3.16.0), and KEGG (Kyoto Encyclopedia of Genes and Genomes, https://www.genome.jp/kegg) pathway enrichment analysis for DEGs was also conducted.

#### 2.4.5 Functional annotation and pathway analysis of DE mRNAs.

DEGs were subjected to GO enrichment analysis using GOseq (release 2.12) and KEGG pathway enrichment analysis using the KOBAS (v2.0) tool and the KEGG database [17]. GO and KEGG pathway enrichments with *p* < 0.05 were considered significant. Appropriate graphs and heatmaps were constructed to show the results.

#### 2.4.6 miRNA isolation, cDNA library construction, sequencing and identification.

Total RNA was isolated from blood in a similar way to the method described for mRNA above, except that small RNAs were separated by size-exclusion chromatography. Specific adaptors were ligated to the 3’ and 5’ ends of the miRNAs, which were converted to cDNA by reverse transcription. The DNA was PCR amplified with primers specific to the adaptor sequences. After the 3’ adaptors and junk sequences were removed to obtain clean sequences, small cDNAs with lengths of 18–26 nt were filtered out, and aligned to the mRNA, RFam, and Repbase databases. The reads that passed length and database filtering were used for miRNA identification with ACGT101-miR (v4.2). Potential miRNAs were identified and analyzed using the miRBase database and the reference genome, and significantly differentially expressed miRNAs (DE miRNAs) were defined as those where *p* < 0.05.

#### 2.4.7 Differentially-expressed miRNA analysis, target gene prediction, and validation.

Target genes of significantly DE miRNAs were predicted using TargetScan (v5.0) [18–20] and miRanda (v3.3a) [21–23]. Target genes with a context score percentile below 50 in TargetScan or maximum free energy (Max Energy)> -10 in miRanda were excluded. The intersection of the two prediction results was taken as the final set of target genes (criteria: TargetScan_score ≥ 50 & miRanda_Energy < -10).

#### 2.4.8 Correlation analysis of DE miRNAs with their DE mRNA targets.

The mRNA target data were screened against the annotated DE miRNAs to predict negative regulatory relationships. Pearson correlation analysis was performed between the normalized counts of DE miRNAs and DE mRNAs for significant negatively correlated DE miRNA/DE mRNA pairs (*p* < 0.05 and r < 0), which were grouped based on the log2 fold-change of the mRNA expression (log2FC > 0; log2FC < 0). Interaction network diagrams were generated using iGraph (version 1.3.0) in R (version 4.0.4) with spherical layouts.

#### 2.4.9 Validation of mRNAs and miRNAs by real-time quantitative PCR (RT-qPCR).

Total RNA was extracted using TRIzol reagent (Invitrogen), following the manufacturer’s instructions. RNA quality was assessed using a Nanodrop spectrophotometer. For cDNA synthesis, 1 μg of RNA was reverse transcribed using the PrimeScript RT Kit (Takara) for mRNA or the TaqMan® MicroRNA Reverse Transcription Kit (Thermo Fisher) for miRNA, following the manufacturer’s guidelines. Quantitative PCR (qPCR) was performed using SYBR® Green PCR Master Mix (Takara) for both mRNA and miRNA. Gene-specific primers for mRNA and miRNA were designed based on known sequences and listed in **S3 Table**. Relative gene expression was calculated using the 2^-^^△△^^CT^method, with GAPDH used as a reference gene for mRNA and U6 for miRNA.

### 2.5 Quantitative lipidomics analysis of blood plasma samples

Blood samples were collected from the jugular vein using 5 mL EDTA anticoagulant tubes, gently mixed by inversion, and centrifuged at 15,000 × g for 10 minutes at 4°C. The supernatant plasma (200 μL) was carefully pipetted into 2 mL cryotubes and stored at -80°C. The plasma samples were analyzed by ultra-performance liquid chromatography (UPLC) (ExionLC™ AD, https://sciex.com.cn/) using a Thermo Accucore™C30 UPLC column (2.6 μm, 2.1 mm × 100 mm i.d.). The solvent system consisted of A: acetonitrile/water (60/40,V/V, 0.1% formic acid, 10 mM ammonium formate), B: acetonitrile/isopropanol (10/90 45 VV/V, 0.1% formic acid, 10 mM ammonium formate); gradient program, A/B 80:20, V/V at 0 min, 70:30 V/V at 2.0 min, 40:60 V/V at 4 min, 15:85 V/V at 9 min, 10:90 V/V at 14 min, 5:95 V/V at 15.5 min, 5:95 V/V at 17.3 min, 80:20 V/V at 17.3 min, 80:20 V/V at 20 min; flow rate, 0.35 ml/min; temperature, 45°C; injection volume: 2 μL. The lipid fractions were identified by tandem mass spectrometry (ESI-MS/MS) an a QTRAP® 6500+ (https://sciex.com.cn/) equipped with an ESI Turbo Ion-Spray interface, operating in positive and negative ion mode and controlled by Analyst 1.6.3 software (Sciex). Lipid contents were detected by MetWare (http://www.metware.cn/) based on the AB Sciex QTRAP 6500 LC-MS/MS platform and comparison with known standards.

#### 2.5.1 Data preprocessing.

Mass spectrometry data were processed using Analyst 1.6.3. The ratio of the integrated peak areas of the detected samples was used to calculate the actual concentration of each substance in the sample. Quality control (QC) samples were included during the detection and analysis to ensure the stability and reliability of the instrument. Pearson correlation analysis was performed on QC samples, and the percentage of compounds with a coefficient of variation (CV) < 0.5 was greater than 85%, indicating stable experimental data. Furthermore, more than 75% of the compounds had a CV < 0.3, consistent with high quality and stability.

#### 2.5.2 Lipid profiling by principal component analysis (PCA).

Unsupervised PCA was performed on the samples to evaluate differences in lipid metabolism between the three groups and the degree of variation within each group using the statistics function prcomp in the R software (www.r-project.org).

#### 2.5.3 Patterns of lipid abundance depicted by clustering analysis.

Lipid content data were processed by unit variance scaling (UVS), and hierarchical clustering analysis (HCA) was conducted to evaluate lipid accumulation patterns across different samples. The sample results and metabolite profiles are presented as heatmaps with dendrograms generated using the ComplexHeatmap package in R software. Pearson correlation coefficients (PCC) between samples were calculated using the cor function in R and visualized as heatmaps. Differential metabolites were determined by two-group analysis with the criteria that VIP > 1 and *p* < 0.05.

#### 2.5.4 Differential lipid metabolite screening by orthogonal partial least squares discriminant analysis (OPLS-DA).

OPLS-DA was applied to the raw data for centering and scaling, using the MetaboAnalystR package and the OPLSR.Anal function in R software. Based on the OPLS-DA model, variable importance in projection (VIP) scores were calculated to preliminarily identify lipids that differed between groups. The criteria for selecting differential metabolites were VIP > 1, *p* < 0.05, and fold change ≥ 2 or ≤ 0.5.

#### 2.5.5 KEGG enrichment analysis of plasma lipid metabolites.

The lipids detected in plasma samples were annotated using the KEGG database, and the differentially expressed metabolites (DEMs) identified were further annotated. Volcano plots of DEMs were generated using the ggplot2 package in R. Heatmaps for hierarchical clustering were plotted using the pheatmap package, with metabolite data standardized using z-scores. Bubble plots were also created using the ggplot2 package, and the KEGG database was used to predict the function of the metabolites and the metabolic pathways they are associated with. Metabolites were considered enriched when x/n > y/n, and pathways were deemed significantly enriched when *p* < 0.05.

### 2.6 Integration of transcriptomics and metabolomics data

PCAs were conducted separately on the transcriptomics and metabolomics datasets. Based on the identified DE genes and metabolites, KEGG pathway enrichment analyses were performed to identify the pathways shared between the two omics datasets.

### 2.7 Statistical analysis

All graphs were generated using GraphPad Prism 8.0 (GraphPad Software Inc, San Diego, CA, USA). Statistical analysis of the data was performed using SPSS 26.0 (IBM, Armonk, NY, USA). Data are expressed as mean ± standard deviation. Differences between groups were analyzed using one-way ANOVA. Homogeneity of variance within groups was tested, with *p* > 0.05 indicating no significant difference.

## 3 Results

### 3.1 Cardiac morphology and functional assessment in Yili horses

**Fig 1:**
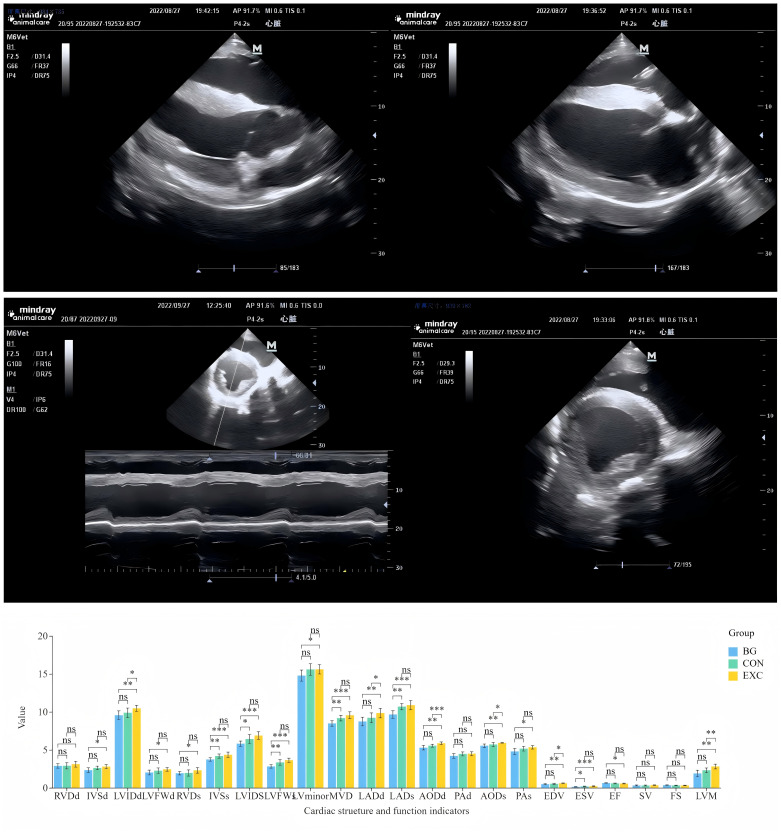
(A) Right parasternal long-axis four-chamber B-mode echocardiogram. (B) B-mode echocardiogram of the right parasternal long-axis left ventricular outflow tract. (C) Right parasternal short-axis B-mode echocardiography. (D) Right parasternal short-axis M-mode echocardiography. (E) Statistical comparisons of echocardiography results from the three performance groups of Yili horses. *p < 0.05, **p < 0.01, ***p < 0.001.

The results of the echocardiographic examinations for heart structure and functional indicators (Fig 1A–C), with comparisons for significance between the groups are shown in Table 1 and Fig 1D.

**Table 1:** Results of echocardiography of the three performance groups of Yili horses under resting conditions showing cardiac structural and functional measurements. BG, no training, n = 6; CON, low performers, n = 10; EXC, high performers, n = 10.

Parameters	BG	CON	EXC
RVDd (CM)	2.92 ± 0.29	2.93 ± 0.41	3.14 ± 0.38
IVSd (CM)	2.36 ± 0.3	2.65 ± 0.24	2.82 ± 0.28
LVIDd (CM)	9.59 ± 0.57	9.91 ± 0.63	10.49 ± 0.4
LVFWd (CM)	2.05 ± 0.32	2.28 ± 0.36	2.46 ± 0.24
RVDs (CM)	1.96 ± 0.22	1.98 ± 0.41	2.33 ± 0.36
IVSs (CM)	3.77 ± 0.22	4.21 ± 0.28	4.38 ± 0.36
LVIDS (CM)	5.85 ± 0.34	6.45 ± 0.59	6.91 ± 0.51
LVFWs (CM)	2.85 ± 0.23	3.38 ± 0.37	3.67 ± 0.28
LVminor (CM)	14.81 ± 0.73	15.61 ± 0.76	15.63 ± 0.61
MVD (CM)	8.5 ± 0.36	9.21 ± 0.37	9.58 ± 0.46
LADd (CM)	8.76 ± 0.57	9.25 ± 0.65	9.87 ± 0.62
LADs (CM)	9.69 ± 0.49	10.72 ± 0.41	10.92 ± 0.6
AODd (CM)	5.32 ± 0.29	5.57 ± 0.2	5.9 ± 0.18
Pad (CM)	4.23 ± 0.29	4.5 ± 0.26	4.52 ± 0.25
AODs (CM)	5.58 ± 0.22	5.74 ± 0.27	5.95 ± 0.04
Pas (CM)	4.82 ± 0.4	5.18 ± 0.3	5.37 ± 0.22
EDV (L)	0.52 ± 0.07	0.56 ± 0.08	0.63 ± 0.05
ESV (L)	0.17 ± 0.02	0.21 ± 0.04	0.25 ± 0.04
EF (%)	0.67 ± 0.05	0.61 ± 0.05	0.60 ± 0.05
SV (L)	0.35 ± 0.06	0.34 ± 0.05	0.38 ± 0.04
FS (%)	0.39 ± 0.04	0.35 ± 0.04	0.34 ± 0.04
LVM (kg)	1.92 ± 0.44	2.34 ± 0.31	2.84 ± 0.32
HR	42.91 ± 3.19	39.49 ± 3.72	38.92 ± 4.8

### 3.2 Transcriptomic changes in Yili horses

#### 3.2.1 DE mRNAs in whole blood samples from the three training groups.

**Fig 2:**
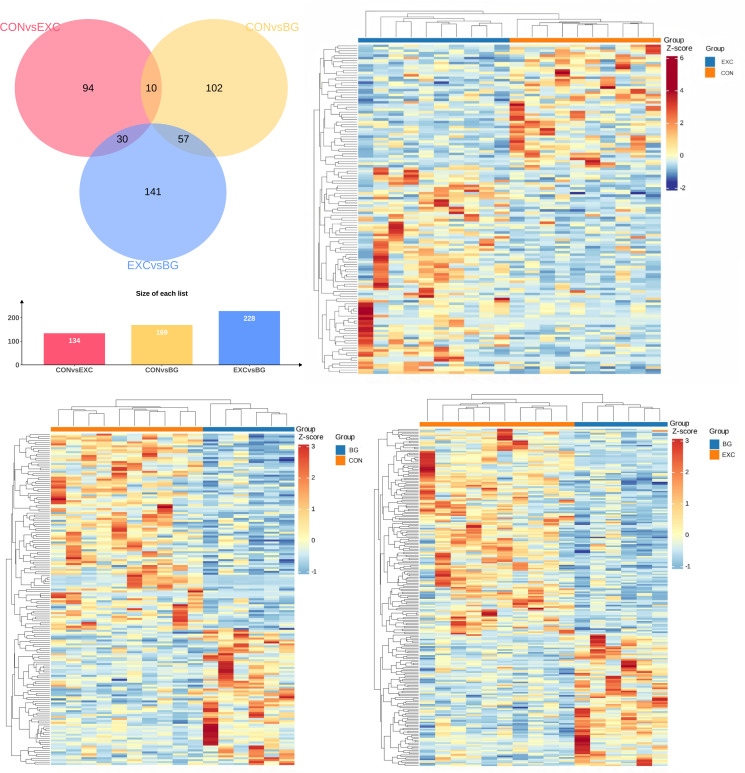
(A) Venn diagram of differentially expressed genes (mRNAs) among the three groups. (B-D) Heatmaps of DEGs in CON vs EXC (B), CON vs BG (C), and EXC vs BG (D).

After sequencing the samples from each group, doing QC, and removing sequencing adaptors and low-quality reads, each sample produced 40.27 to 51.91 million clean reads, accounting for over 98% of the original reads. The sequencing error rate was below 0.01%, and the GC content ranged from 50.41% to 54.92%. The clean reads were aligned with the reference horse genome, https://ftp.ncbi.nlm.nih.gov/genomes/all/GCF/002/863/925/GCF_002863925.1_EquCab3.0/, and corresponding genes were identified with HISAT2 [1]. A total of 48,303 genes were identified across the three groups, and Fig 2A shows the numbers of DE mRNAs overlapping among the three groups in a Venn diagram. Based on the differential screening criteria, 534 DEGs were identified (Fig 2A). Between the CON and EXC groups, 134 DEGs were identified, with 51 genes upregulated and 83 downregulated. Between the EXC group and the BG group, 228 DEGs were identified, with 138 upregulated and 90 downregulated. Between the CON group and the BG group, 169 DEGs were identified, with 100 upregulated and 69 downregulated. The top-performing group (EXC) had the most DEGs compared with the blank group (BG). The DEGs were subjected to hierarchical clustering analysis to display the gene expression changes, and heatmaps of clustered DEGs for each group are shown in Fig 2B–D. The individuals within each group showed good within-group clustering, while significant differences were observed between groups.

#### 3.2.2 Functional classification and annotation of DE mRNAs.

**Fig 3.**
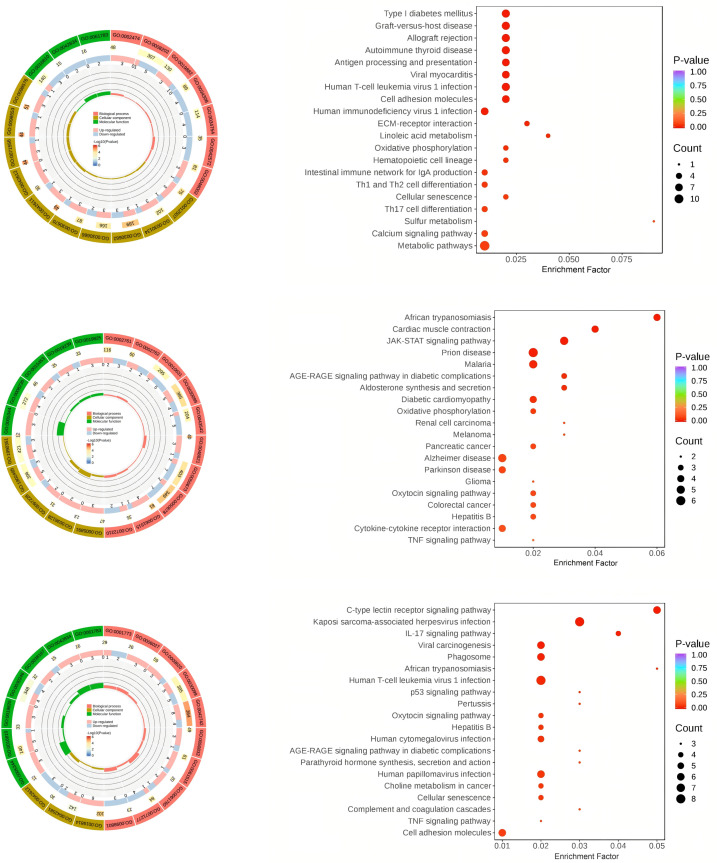
(*upper*) GO chord diagrams for CON vs EXC (A), CON vs BG (C), and EXC vs BG (E). (*lower*) KEGG bubble plots for CON vs EXC (B), CON vs BG (D), and EXC vs BG (F).

To clarify the biological functions and classifications of the DEGs, GO analysis was performed on the DE mRNAs identified in each group (Fig. 3A–C). The DEGs between the CON and EXC groups were primarily enriched in biological processes such as peptide antigen processing and presentation, cellular components like the integral component of the luminal side of the endoplasmic reticulum membrane, and molecular functions involving peptidoglycan binding. In the CON vs BG groups, the DEGs were mainly associated with erythrocyte development, the sperm flagellum as a cellular component, and oxygen carrier activity as a molecular function. Hemoglobin binds to oxygen in the lungs and transports it to the muscles and to the heart itself, so the better the oxygen carrier activity, the greater a horse’s endurance and stamina will be, especially during a race [24]. Oxygen also promotes recovery and healing of muscles stressed from intense activity, which would also be beneficial where animals need to recover full power before the next performance event. Regular exercise improves the horse’s cardiovascular system, increasing its capacity for delivering oxygen to the muscles [25]. Similarly, DEGs between the EXC and BG groups were enriched in biological processes related to defense responses to bacteria, cellular components involving the immunoglobulin complex, and molecular functions such as oxygen carrier activity. KEGG analysis of the DEGs (Fig 3D–F) indicated significant enrichment across pathways related to environmental information processing, organismal systems, cellular processes, and metabolism.

Compared to the CON group, the DEGs in the EXC group were enriched in pathways involved in oxidative phosphorylation, metabolic processes, and calcium signaling. The normal functioning of the heart depends on various signaling pathways, including those associated with metabolism of glucose, fatty acids, and amino acids. These pathways provide the materials and energy for cardiomyocyte activity [26]. Cardiomyocytes require a large amount of energy to maintain normal contraction and relaxation functions, with oxidative phosphorylation being the main metabolic pathway for intracellular energy (ATP) production [27]. Exercise training, especially endurance training, can increase the number and size of mitochondria in muscles providing horses with a greater capacity for sustained energy output, as during a race [28].

A regular training program increases the activity of enzymes involved in both aerobic and anaerobic ATP production, which means that the horse’s muscles can more efficiently convert a variety of fuel sources such as lipids into energy. Horses have different types of muscle fibers, and exercise training can cause a shift in the proportion of these fibers, favoring those with a higher capacity for ATP production. For example, an increase in type I and type IIa fibers, which are more oxidative and fatigue-resistant. Young horses may be more likely to show a shift towards type IIa fibers with training, while older horses may have less capacity for fiber type transformation. Different muscles in the horse’s body may show different adaptations to exercise [29].

The DEGs between the CON group and the BG group are mainly enriched in the JAK-STAT signaling pathway, cardiac muscle contraction, and the TNF signaling pathway. These pathways are involved in signal transduction processes that play significant roles in cardiovascular diseases [30]. Studies in humans and mice have shown that the JAK/STAT pathway is involved in cardiac remodeling associated with heart failure, myocardial infarction, and hypertension. It can contribute to hypertrophy (enlargement of the heart), fibrosis (scarring of heart tissue), and inflammation [31]. The DEGS between the EXC group and the BG group are enriched in the p53 signaling pathway, cellular senescence, and the TNF signaling pathway, which have critical impacts on cardiomyocyte apoptosis and cardiac hypertrophy [32].

#### 3.2.3 Analysis of DE miRNAs in Yili horse blood.

**Fig 4:**
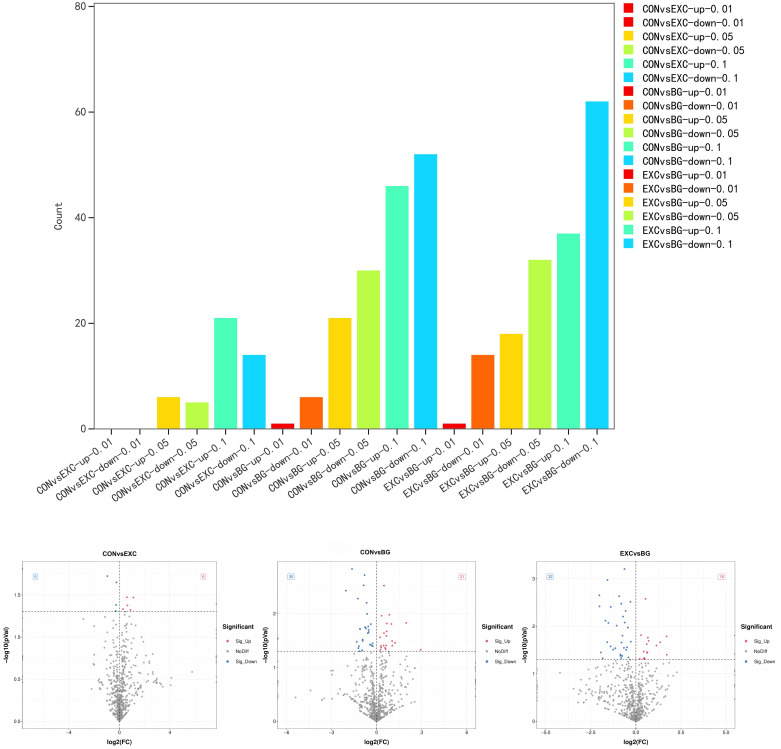
(*upper*) Results of miRNA-Seq for each group of Yili horses. **(A)** Upregulated and downregulated DE miRNAs among the three groups. (*lower*) Scatter plots for CON vs EXC **(B)**, CON vs BG **(C)**, and EXC vs BG **(D)** with the top ten most significant DE miRNAs labeled. The scatter plots illustrate the upregulation and downregulation of DE miRNAs between the different groups: **(B)** CON vs EXC, **(C)** Con vs BG, and **(D)** EXC vs BG.

A total of 2,428 miRNAs were identified, including 366 DE miRNAs, with 46 DE miRNAs detected between the CON and EXC groups (27 upregulated and 19 downregulated), 156 DE miRNAs detected between the CON and BG groups (68 upregulated and 88 downregulated), and 164 DE miRNAs detected between the EXC and BG groups (56 upregulated and 108 downregulated). To verify the accuracy of the sequencing data, we randomly selected several miRNAs for validation by qPCR and the results were consistent with the miRNA-seq data (Fig 4).

#### 3.2.4 Association analysis of DE miRNAs and their DE target genes.

**Fig 5:**
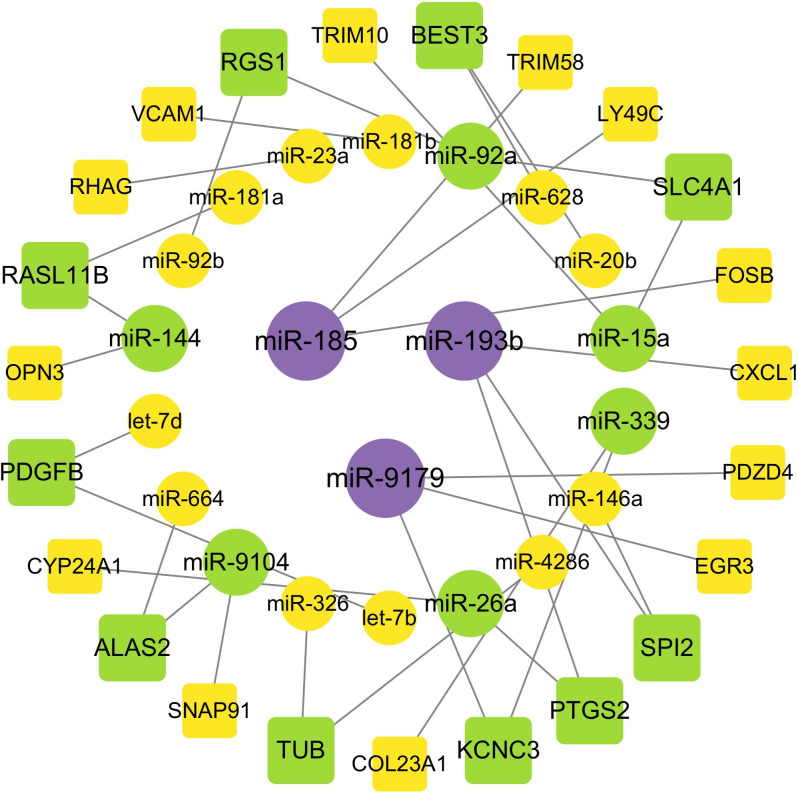
Network of interactions between miRNAs and their target genes. The circles are miRNAs, and the squares are the targets.

By utilizing both the TargetScan (v5.0) and miRanda (v3.3a) databases, we cross-referenced the predicted target genes with the mRNA transcriptome obtained through RNAseq. A total of 33 negatively co-expressed pairs were identified (Fig 5). Screening for mRNA-miRNA interactions yielded the following pairs of target/miRNAs: *PTGS2*/miR-193b, -26a; *OPN3*/miR-144; *SLC4A1*/miR-15a, -92a; *CYP24A1*/miR-26a; *TRIM10*/miR-15a; *RGS1*/miR-92a; *SPI2*/miR-146a,-193b; *RAS11B*/miR-181a,-144; *VCAM1*/miR-181b; *RHAG*/miR-23a; *TRIM58*/miR-185; *TUB*/miR-326,-4286; *PDZD4*/miR-9179; *LY49c*/miR-185, *ALAS2*/miR-664,-9104; *KCNC3*/miR-9179,-339; *FOSB*/miR-185, *PDGFB/*let7b, 7d; *EGR3*/miR-9179; *BEST3*/miR-20b,-628; *SNAP91*/miR-9104; *CXCL1*/miR-193b; and *COL23A1*/miR-339. The gene/miRNA combinations, *ALAS2*/miR664,-9104*, BEST3*/miR-20b,-628*, KCNC3*/miR-9179,-339*, PDGFB*/let7b,7d, *RASL11B*/miR-181a,-144, and *VCAM1*/miR-181b, have been shown to regulate cardiac function.

### 3.3 RT-qPCR validation

**Fig 6:**
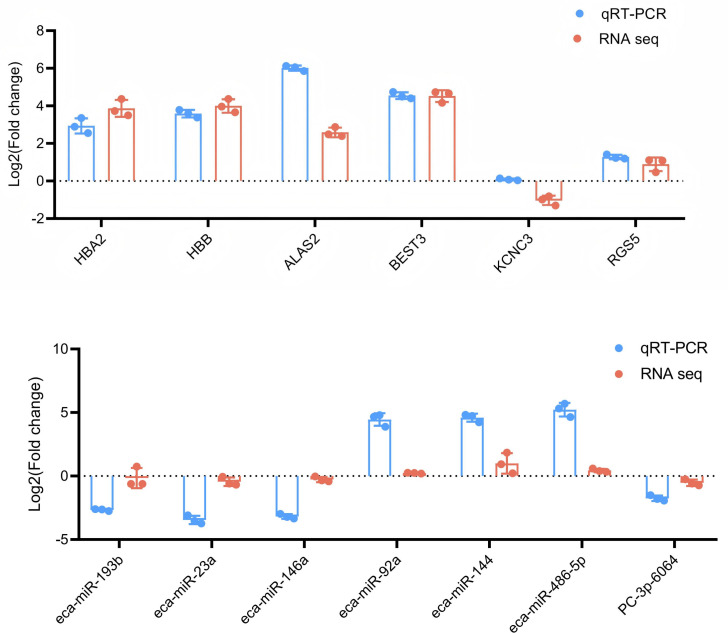
The results of RNA-seq analysis (A) mRNA; (B) miRNA.

**Fig 7:**
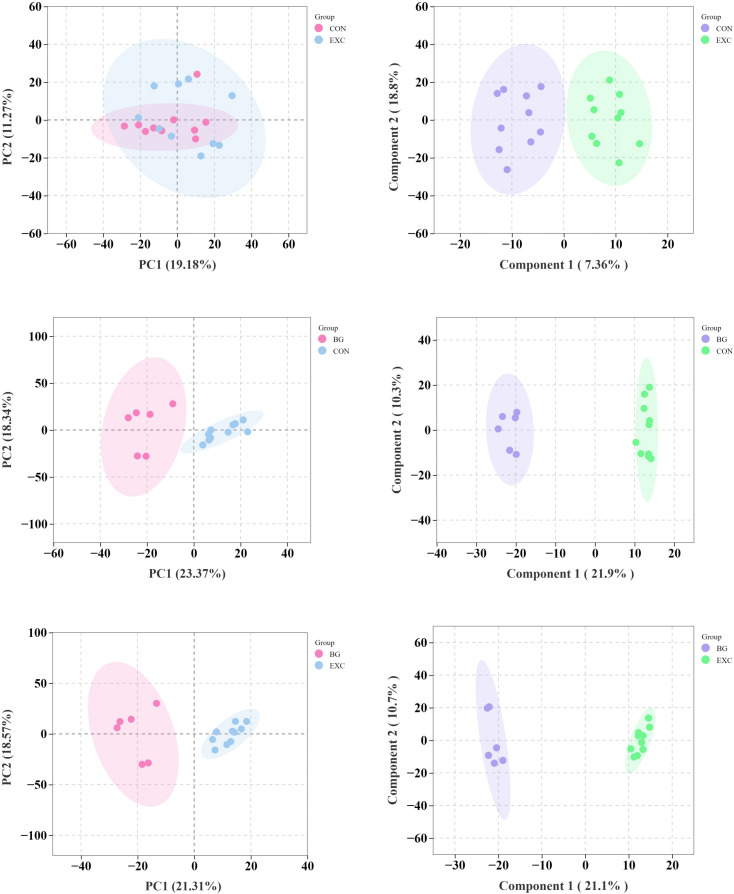
(A) PCA plot of CON and EXC. (B) OPLS-DA scores for CON and EXC. (C) PCA plot of CON and BG. (D) OPLS-DA scores for CON and BG. (E) PCA plot of EXC and BG. (F) OPLS-DA scores for EXC and BG.

To determine the accuracy of sequencing data, 6 mRNAs (Fig 6A) and 7 miRNAs (Fig 6B) were randomly selected for RT-qPCR analysis. As shown in Fig 6, the expression levels and change trends of 6 genes and 7 miRNAs in the blood of horses of different breeds were basically consistent with the results of RNA-seq analysis, indicating that the RNA-seq sequencing data in this experiment were accurate and could be used for further research and analysis.

### 3.4 Metabolomic changes in Yili horses resulting from performance training

#### 3.4.1 Analysis of differentially expressed metabolites in plasma.

We used lipidomics technology with data from UPLC-MS/MS quantitation and identified 1373 metabolites. PCAs were performed on all experimental and quality control (QC) samples to preliminarily assess the degree of variation among samples (Fig 7A, C, E). Differences were observed between samples, with the degree of separation between the CON and EXC groups being moderate, indicating certain shared metabolic characteristics between horses with similar exercise regimens differing primarily in intensity. The separation between the CON and BG groups was significant, and the separation between the EXC and BG groups was even more pronounced. All samples fell within the elliptical confidence interval, showing clear distinctions between the different groups.

The results of the OPLS-DA analysis demonstrated that the R² values were greater than the Q² values when the model was sorted. This indicates that the explanatory power of the model exceeded its predictive power, confirming that the model was not overfitted. The model was stable, reliable, and capable of accurately describing the samples (Fig 7B, D, F).

#### 3.4.2 Differences in lipid metabolites across groups.

**Fig 8:**
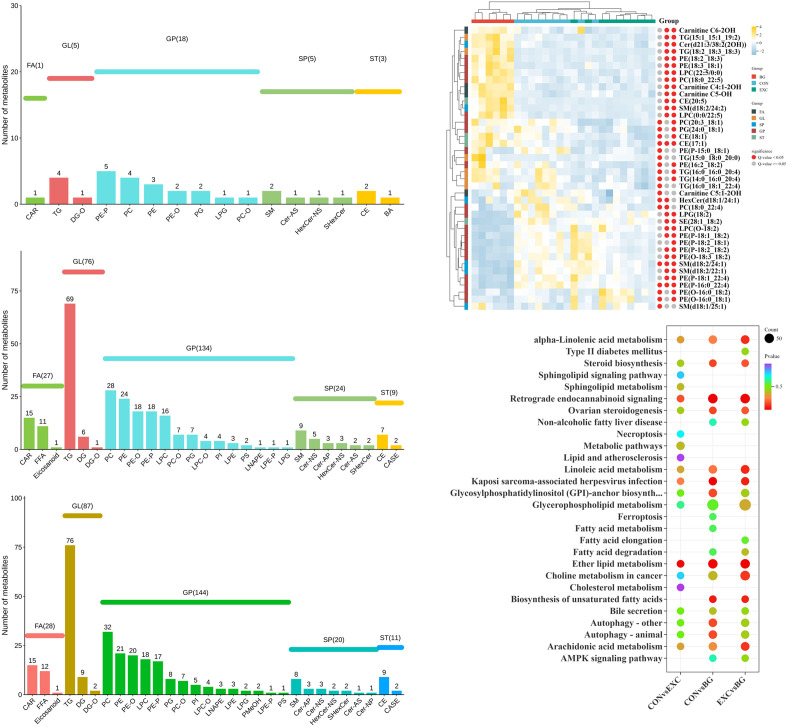
Lipid metabolite classification differences among the three groups. **(A)** CON and EXC comparison. **(B)** BG and CON comparison. **(C)** BG and EXC comparison. **(D)** Heatmap of differential metabolites among the three groups. **(E)** Pathway comparisons for the differentially expressed metabolites of each group.

Based on the variable importance in projection (VIP) values obtained from the OPLS-DA model, we identified metabolites with VIP > 1 and *p* < 0.05. A total of 592 DE metabolites were identified and classified into five major categories and 33 subcategories. **CON vs EXC:** 32 differential metabolites were identified, with 26 upregulated and 6 downregulated. **EXC vs BG:** 317 differential metabolites were identified, with 105 upregulated and 212 downregulated. **CON vs BG:** 346 differential metabolites were identified, with 175 upregulated and 171 downregulated (**Fig. 8A-C**).

By filtering metabolites with higher VIP values and relevance to the heart and exercise, it was observed that the differences between the CON and EXC groups were minimal and primarily concentrated in the GP subcategory. In contrast, the significant differences in lipids between the two training groups (EXC and BG) were similar and concentrated in the TG and PC subcategories (Fig 8D). This suggests that the effects of training on horses are mainly focused on the GL and GP categories, warranting further research.

KEGG enrichment analysis indicated that the differential metabolites between the CON and EXC groups were primarily enriched in ether-lipid metabolism, retrograde endocannabinoid signaling, and Kaposi sarcoma-associated herpesvirus infection (Fig 8E). For **CON vs BG**, the regulated metabolites were mainly involved in Kaposi sarcoma-associated herpesvirus infection and Retrograde endocannabinoid signaling. For **EXC vs BG**, similar enrichments were observed in ether-lipid metabolism, Retrograde endocannabinoid signaling, and Kaposi sarcoma-associated herpesvirus infection.

### 3.5 Integrated analysis of transcriptomics and metabolomics

**Fig 9:**
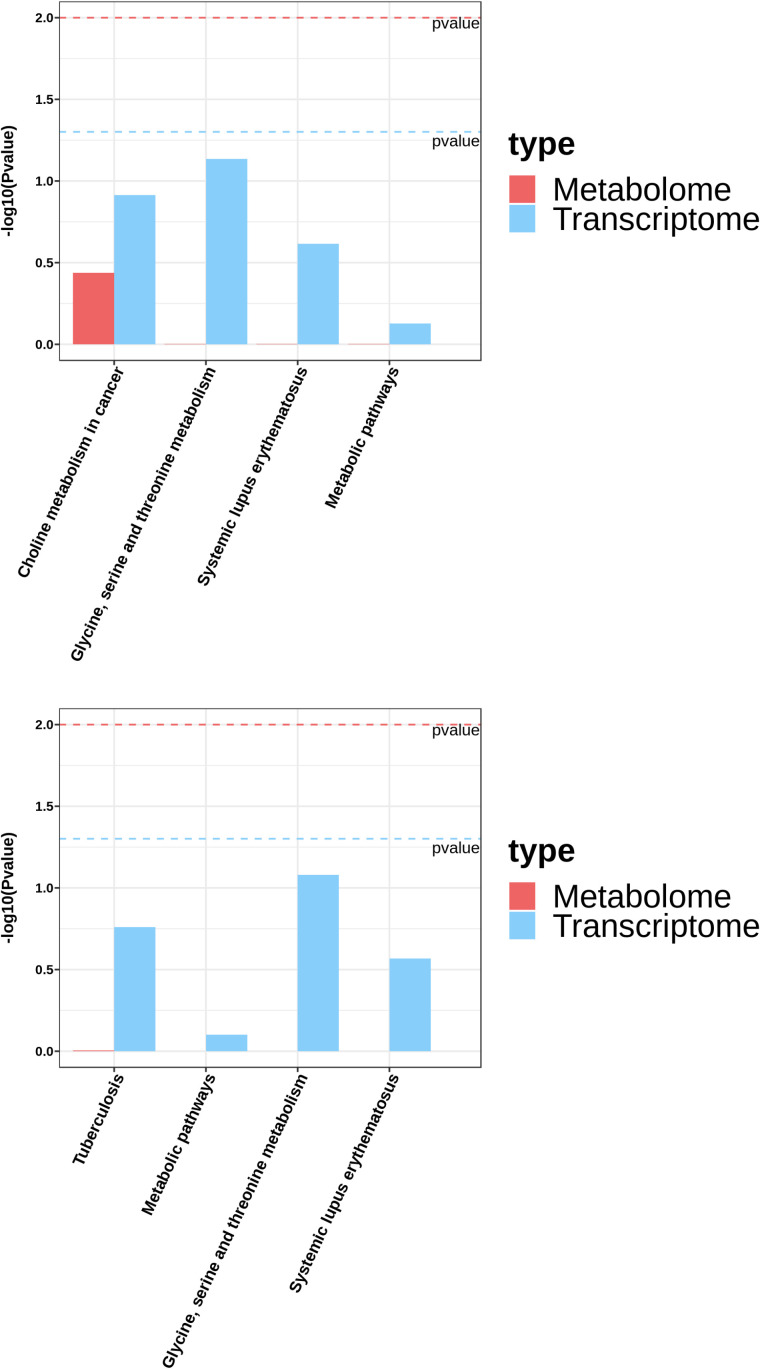
Integrated analysis of data from transcriptomics and metabolomics analyses: CON vs BG (A), EXC vs BG (B).

To determine the differences in biological pathways of average performance horses compared with high performance horses, we integrated the results of transcriptomics and metabolomics. A histogram was plotted using KEGG pathway enrichment data from the two bioinformatic screenings, which measured the degree of association between DEGs and DEMs according to the *p*-value of enrichment in selected pathways. The KEGG database was then used to analyze the integrated pathways of DEGs and DEMs from transcriptome and metabolome analysis (Fig 9). Compared with the BG, the main pathways of significant enrichment of the CON group involved glycine, serine and threonine metabolism, choline metabolism in cancer, and systemic lupus erythematosus. Compared with the BG, the metabolic pathways of the EXC group had a greater prevalence of glycine, serine and threonine metabolism, tuberculosis, and systemic lupus erythematosus. No pathway enrichments were found in the CON compared with the EXC groups.

## 4 Discussion

Recent studies have shown a significant correlation between heart size and athletic performance in horses. Particularly for racehorses, the size and structural characteristics of the heart are considered important factors influencing their competitive performance. For example, Thoroughbred racehorses typically have larger heart volumes, which helps them maintain higher oxygen uptake and cardiac output during intense exercise [33]. Furthermore, different types of physical activity exert varied effects on the equine heart [34]. This indicates that cardiac development is influenced not only by genetic factors but also by postnatal training and the type of physical activity. The relationship between the structure and function of the equine heart and athletic performance is complex with many factors involved. Future research needs to explore the molecular mechanisms of cardiac developments in relation to performance and how genetic and environmental factors interact to optimize athletic performance in horses. This has significant implications not only for the horse racing industry but also for providing new perspectives and methods for cardiac research in other animals.

The term ‘cardiac remodeling’ was originally used to refer to changes at the cellular and molecular levels caused by pathological stimuli like infarction, but has now been broadened to include alterations in heart size, shape, and function resulting from normal physiological changes such as performance training, which is how the term is used here [35,36].

Endurance training leads to overall cardiac enlargement, primarily due to increased left ventricular mass. The left ventricle is responsible for pumping oxygen-rich blood throughout the body. After three months of desensitization and another three months of regular training, the LVIDd and LVIDs values of the training group horses were significantly larger than those of the untrained group. A study on human athletes found that endurance training led to structural changes in the left ventricle, characterized by an eccentric hypertrophy pattern, where chamber dilation was accompanied by a relative increase in wall thickness. In contrast, resistance training may lead to concentric hypertrophy, characterized by increased ventricular wall thickness and greater overall mass. Endurance training primarily affects the left ventricle, adapting it to meet the demands of high-intensity exercise [37]. Although there were no differences in LVIDd between the CON and BG groups, there were significant differences in LVIDs. Exercise enhances cardiac function by improving contractility and efficiency, enabling the heart to pump more blood with less effort. This means better oxygen delivery to muscles, including the heart itself, during exertion [38]. Consequently, while the CON group did not show significant differences in LVIDd, their stroke volume (the amount of blood pumped per beat) was higher than that of the non-trained BG group.

As mentioned above, the shape of the left ventricle reflects the type of exercise. Endurance training in racehorses often results in a more elongated left ventricle [39]. After just six months of training, we observed that horses with stronger athletic performance had larger LVminor dimensions. Long-term training-induced changes in left ventricular structure involve multiple mechanisms, with significant alterations in myocardial mechanics due to stretching and microstructural changes of myocardial fibers. Left ventricular dilation and hypertrophy are adaptive responses to the increased blood flow load frequently encountered during high-intensity exercise [40]. Training also leads to significant enlargement of the left atrial volume [41], a phenomenon observed in our echocardiographic comparisons among the three groups. In this study, the aortic diameter of the training group was significantly larger than that of the untrained group, which deserves attention. An echocardiographic study of 3,781 athletes found that the aortic diameter in athletes was slightly larger than in the control group. The aorta is a core component of the circulatory system, ensuring the effective delivery of oxygen and nutrients to various parts of the body to meet the physiological demands of increased exercise [42].

The effects of exercise on the myocardium and vascular system depend on the frequency, intensity, and duration of the exercise itself. In previously untrained individuals, long-term (≥6 months) regular high-intensity exercise typically resulted in a decrease in resting heart rate and submaximal load heart rate by 5–20 beats per minute, along with enhanced myocardial contractility. Structurally, the volumes of all four cardiac chambers increase, with a slight increase in wall thickness, leading to greater heart mass due to the enlargement of myocardial cells [43]. In this study, we observed a similar pattern of differences across the three groups: the horses with the strongest athletic performance had the largest left ventricular volumes and lowest resting heart rates.

### 4.1 Profiling and comparison of mRNA levels in the blood of Yili horses at high vs low performance levels

Changes in mRNA expression play a crucial role in the body’s response to exercise and disease. In horses, exercise training alters the expression of genes involved in muscle growth, energy metabolism, and inflammation, and analyzing transcriptomics profiles can help to customize training programs for individual horses and breeds depending on the type of athleticism desired.

We identified a total of 534 differentially expressed genes (DEGs). Among them, the genes related to erythrocyte regulation showed higher expression levels in the EXC vs BG group (e.g., ALAS2: log2FC = 4.26, HBA: log2FC = 3.93, RHAG: log2FC = 4.93, SLC4A1: log2FC = 3.07, TRIM58: log2FC = -1.12, HBB: log2FC = 3.57, HBA2: log2FC = 3.39) and in the CON vs BG group (e.g., ALAS2: log2FC = 4.29, HBA: log2FC = 3.93, SLC4A1: log2FC = 2.94, HBB: log2FC = 3.67, HBA2: log2FC = 3.75).

Erythrocytes are the most abundant cell type in the body, and their primary physiological function is to transport oxygen to tissues and organs through the circulatory system and CO_2_ back to the lungs. The development of erythrocytes involves a process where multipotent hematopoietic stem cells proliferate and differentiate into mature red blood cells. This process includes unique changes such as cell size reduction, increased hemoglobin synthesis, chromatin condensation, and enucleation.

ALAS2 encodes 5’-aminolevulinic acid synthase-2, which is the enzyme that performs the first step in heme biosynthesis. Heme deficiency can cause severe problems in the cardiovascular system [44]. In addition to the hemoglobin in the blood, the heme group is required for the synthesis of myoglobin, which is the heme protein in muscles that stores oxygen for use during muscle activity. Thus, having sufficient levels of ALAS2 is critically important for racing performance in horses.

The expression of HBB2 and HBG2 plays a key role in the development of erythrocytes [45]. Additionally, the regulation of HBA2 and HBB expression is a vital step in erythropoiesis, especially in adults, as the balance between alpha-globin and beta-globin is crucial for normal red blood cell function. Studies have shown that the expression level of HBA2 is closely associated with erythrocyte functionality, and its variation may affect individual hemoglobin levels and red blood cell physiology [46–48].

Through gene ontology (GO) enrichment analysis, we found that the differentially expressed genes (DEGs) in both the EXC vs BG and CON vs BG groups were annotated to the biological process term, erythrocyte development’. KEGG pathway enrichment analysis revealed that the DEGs in the CON vs. EXC group were enriched in pathways such as viral myocarditis, ECM-receptor interaction, and oxidative phosphorylation.

Viral myocarditis is a disease caused by viral infection of cardiomyocytes, where the immune response triggered by the infection directly damages myocardial tissue, leading to cardiomyocyte necrosis, inflammatory cell infiltration, myocardial dysfunction, arrhythmias, and heart failure. Studies have shown that in the ECM-receptor interaction pathway, the binding of integrin receptors on cardiomyocytes and fibroblasts to the extracellular matrix transmits mechanical signals, thereby regulating the functions and phenotypes of these cells. During cardiac contraction, the mechanical properties of the ECM, such as elasticity modulus and viscosity, change. These changes affect myocardial contractility and cardiomyocyte proliferation[49]. Cardiomyocytes require large amounts of energy to maintain normal contraction and relaxation functions, and oxidative phosphorylation is the primary intracellular metabolic pathway for producing energy (ATP). Through this process, mitochondria in cardiomyocytes provide energy to sustain myocardial contraction/relaxation. During exercise, energy demands increase substantially across tissues, and oxidative phosphorylation plays a critical role in supplying energy for muscle contraction and maintaining normal cardiac function during physical activity.

The DEGs in the CON vs. BG group were enriched in the cardiac muscle contraction pathway, which involves multiple molecules and signal transduction processes. The molecular mechanisms underlying myocardial contraction include the proper functioning of calcium release channels, as well as the dynamics, physiological regulation, and pathological changes of voltage-gated calcium channels that induce calcium release. These processes are fundamental to the heart’s pumping action [50]. The JAK-STAT signaling pathway plays a critical role in many aspects of cardiac physiology and pathology, including ischemia, ischemic preconditioning, and remote preconditioning, where it exhibits protective effects [51].

The DEGs in the EXC vs BG group were enriched in the p53 signaling pathway, which plays a complex and critical role in cardiac health and disease. This pathway is involved in cardiac function, cardiomyocyte apoptosis, cardiac fibroblast senescence, myocardial fibrosis, and the progression of cardiovascular diseases.

Both the trained and the non-trained groups were enriched in the TNF signaling pathway. Research has shown that intense endurance exercise can lead to atrial structural remodeling in mice, which is dependent on TNFα activation. Post-exercise, phosphorylation levels of downstream factors in the TNFα signaling pathway, such as NF-κB, increase, leading to atrial fibrosis and heightened susceptibility to atrial fibrillation (AF). Additionally, using TNFα inhibitors or TNFα-knockout mice significantly reduced exercise-induced AF and atrial fibrosis [52].

### 4.2 Regulation of circulating miRNAs in Yili horses

In this study, the miRNA target genes predicted to overlap with genes identified through transcriptome sequencing were screened, and a total of 33 negative-regulatory miRNA-mRNA pairs were identified. We found that miR-9179 and miR-339 jointly regulate KCNC3, which encodes a potassium ion channel that may play an important role in neuronal regulation of heart rhythms. Let7b and let7d jointly target and regulate the PDGFB gene, which encodes platelet-derived growth factor B that promotes cell growth, proliferation, and angiogenesis, processes necessary for tissue repair and regrowth. MiR-181b regulates VCAM1, which encodes vascular cell adhesion molecule 1. This protein is expressed in the vascular endothelium of arteries and serves as a binding site for leukocytes in response to inflammation, potentially contributing to the formation of atherosclerotic plaques.

In the CON vs. EXC group, miRNAs primarily regulated genes associated with vitamin D metabolism, neuronal signaling, and structural support of bone and cartilage. In the EXC vs. BG group, several miRNAs were significantly differentially expressed and closely associated with cardiac remodeling.

In this study, miR-23a was found to promote erythrocyte growth by regulating RHAG, which is involved in erythrocyte development. This finding is consistent with a study on resistance training, which suggested that miR-23a might play a role in vascular development [53].

In both the EXC vs. BG and CON vs. BG groups, we identified miR-92a as a regulator of the RGS1 gene. MiR-92a targets RGS3, KLF2, and GDF11, regulating endothelial cell proliferation and inflammatory responses by inhibiting endothelial cell growth and angiogenesis. Both RGS1 and RGS3 suppress signal transduction through S1P(1), S1P(2), and S1P(3) receptors, which play essential roles in the cardiovascular system [54]. In the EXC vs. BG group, miR-92a and miR-15a were identified as regulators of SLC4A1, which is critical for maintaining erythrocyte function and regulating bicarbonate and chloride ion exchange [55].

MiR-15a suppresses cardiac hypertrophy and fibrosis by inhibiting the TGF-β signaling pathway [56]. In this study, miR-15a was found to regulate TRIM10, which encodes a protein containing three zinc-binding domains (a RING, a B-box type 1, and a B-box type 2), and a coiled-coil region. This protein is localized to the cytosol. A mouse study demonstrated that this protein plays a role in the terminal differentiation of erythrocytes [57].

In the EXC vs BG groups, miR-26a and miR-193b were found to regulate PTGS2, which is involved in pathways such as energy production by oxidation of organic compounds and oxidoreductase activity acting on single donors with incorporation of molecular oxygen, O_2_. These pathways play a critical role in cardiac energy metabolism and are essential for maintaining normal cardiac cell function and overall heart health. Additionally, miR-26a regulates CYP24A1, which is involved in biological processes such as the fatty acid metabolic process and lipid catabolic process, providing energy for the heart. MiR-181a and miR-144 regulate RASL11B, which participates in myocardial infarction, atherosclerosis, cardiac enlargement (ventricular hypertrophy), and heart failure through TGF-β1-mediated pathways [58,59]. MiR-144 also affects cardiac remodeling by targeting components of the PI3K/AKT/mTOR signaling pathway, such as Pik3a, Pten, and Tsc2 [60]. MiR-146a mitigates myocardial ischemia-reperfusion (I/R)-induced cell damage by inhibiting the NOX4/P38 signaling pathway [61].

### 4.3 Regulation of lipid composition and content in Yili horse blood

The role of lipid compounds in cardiac remodeling is a complex and multi-faceted process. As crucial biomolecules, blood lipids not only play key roles in energy storage and cell structure, but are also closely associated with various physiological and pathological processes [62]. Lipids in the blood include cholesterol, triglycerides, and phospholipids, all of which are essential for maintaining normal cell structure and function.

In the CON and EXC groups, lipid compounds were primarily represented by the PE-P subclass, while differential lipid compounds between the trained and untrained groups were aggregated in the TG subclass. Across broader categories, the greatest differences in lipid compounds among the three groups were observed in the GP category. After screening the differential lipid compounds based on VIP scores and their relevance to cardiac function, it was found that TG(16:0_16:0_20:4), TG(14:0_16:0_20:4), TG(16:0_18:1_22:4), and TG(15:0_18:0_20:0) were significantly expressed in the CON and EXC groups. Triglycerides (TGs) are one of the primary lipid components in blood, and elevated levels are associated with increased cardiovascular disease risk. The lipidomics results for the trained and untrained groups shared some similarities, with a high frequency of acylcarnitines. Acylcarnitines facilitate the transport of fatty acids from the cytoplasm to the mitochondria for β-oxidation, and changes in acylcarnitine levels produced during fatty acid oxidation may reflect alterations in cardiac energy metabolism. Carnitine derivatives such as C4:1–2OH and C5-OH could regulate fatty acid metabolism and energy production, which is particularly important for the heart, an organ with high energy demands [63].

The results of KEGG analysis revealed that lipid compounds in the CON and EXC groups, as well as those in the EXC and BG groups, were enriched in the ether lipid metabolic pathway. Ether lipids are a unique class of glycerophospholipids characterized by an ether bond linking an alkyl chain to the sn-1 position of the glycerol backbone. They account for approximately 20% of the total phospholipid pools in mammals and are especially abundant in the brain, heart, spleen, and leukocytes. Plasma phospholipids are the most abundant form of ether lipids in the body. They are rich in polyunsaturated fatty acids (PUFAs), including docosahexaenoic acid (DHA) and arachidonic acid (AA), which are often located at the sn-2 position [64]. The differential lipids in the CON vs. BG and EXC vs. BG groups were significantly enriched in the retrograde endocannabinoid signaling pathway. Retrograde endocannabinoid signaling is a neuroendocrine system that regulates energy balance, as well as lipid and glucose metabolism, processes closely associated with cardiovascular risk [65].

### 4.4 Integrated analysis of transcriptomics and metabolomics in Yili horses

Although the CON and EXC groups were not co-enriched in the same pathways, comparisons across the different datasets revealed that exercise performance influenced the expression of specific genes and metabolites, including those under control of DE miRNAs. In the trained groups, we found enrichment in the glycine, serine, and threonine metabolic pathways, which play a crucial role in athletic performance, undergoing significant changes in response to exercise. These changes affect various aspects of performance, including energy production, muscle growth and repair, and fatigue resistance. Glycine is necessary for the synthesis of hemoglobin, a key component of hemoglobin, which is essential for the maturation and function of erythrocytes, and in the process of hemoglobin synthesis one iron atom and eight glycine molecules are required for each hemoglobin molecule synthesized [66]. Glycine is a key component of creatine, which is essential for rapid energy production in muscle cells [67]. Increased creatine availability can enhance short-duration, high-intensity performance. Glycine also serves as a critical precursor for numerous biosynthetic reactions, such as protein synthesis. Glycine, serine, and threonine are all important building blocks for protein synthesis and adequate availability of these amino acids supports muscle growth and repair of tendons and ligaments after exercise [68]. Initially utilized by the colonic microbiota as an energy source, glycine is subsequently metabolized in the liver into glycogen and uric acid, which contribute to the body’s energy reserves [69]. During intense exercise, ammonia accumulates as a byproduct of protein breakdown, and glycine participates in ammonia detoxification in the liver, which reduces fatigue and improves exercise tolerance.

Serine, a non-essential amino acid, plays a key role in cell proliferation and survival [70]. It is also a precursor of the antioxidant compound glutathione, which lowers oxidant levels produced by exercise and reduces muscle stress [71]. Regulation of serine metabolism is essential for maintaining cellular redox homeostasis and lipid metabolism [72]. A combined analysis using radiolabeled tracers and a cardiac metabolism model (CardioNet) revealed that serine supplementation significantly enhances the rate of cardiac protein synthesis, whereas threonine had no significant effect. This suggests that serine plays a crucial role in cardiac protein synthesis, potentially contributing to the maintenance of cardiac function and adaptive changes. [73].

Threonine metabolism may also indirectly affect oxidative phosphorylation processes by influencing mitochondrial function and lipid metabolism. [69,74,75].

In summary, the glycine, serine, and threonine metabolic pathways are closely tied to athletic performance, and exercise induces significant changes in these pathways that can contribute to energy production, muscle growth and repair, and fatigue resistance.

Although the integrated analysis of transcriptomics and metabolism yielded limited results, we were still able to identify some connections from the data. Oxidative phosphorylation generates mitochondrial membrane potential, which is used to synthesize ATP and drive the transport of mitochondrial proteins and metabolites. During erythrocyte development, oxidative phosphorylation may provide the energy required for the growth and differentiation of red blood cells. As the primary energy production process in cells, oxidative phosphorylation is especially critical for the heart, a high-energy-demanding organ. Transcriptomic sequencing revealed that miR-9179, miR-339, miR-181b, miR-23a, miR-92a, miR-15a, miR-26a, and miR-193b regulate genes such as KCNC3, PDGFB, VCAM1, RHAG, RGS1, SLC4A1, TRIM10, CYP24A1, and PTGS2, which are involved in the growth and development of erythrocytes.

The production of ATP in cardiomyocytes primarily relies on oxidative phosphorylation of fatty acids (40–60%) and, to a lesser extent, on glucose oxidation or glycolysis. Triglycerides (TGs) serve as the storage form of free fatty acids (FFAs) and are present in lipid droplets within cardiomyocytes [76]. When needed, TGs can be hydrolyzed by lipases into FFAs and glycerol, releasing FFAs for energy production.

Phospholipids play a crucial role in cellular biological membranes, particularly in the inner mitochondrial membrane. The main phospholipid component of the inner mitochondrial membrane is cardiolipin (CL), which not only provides the necessary membrane environment for the proteins and enzymes of the electron transport chain (ETC) but also plays an essential role in maintaining mitochondrial structure and function [77].

Lipidomics results also indicated that TGs and PCs (phosphatidylcholines) exhibited the highest abundance. MiR-193b and miR-26a regulate PTGS2, which participates in oxidative phosphorylation. KEGG analysis also identified significant enrichment of DEGs in the oxidative phosphorylation pathway. Moreover, miR-26a regulates CYP24A1, which is involved in lipid metabolism.

**Fig 10:**
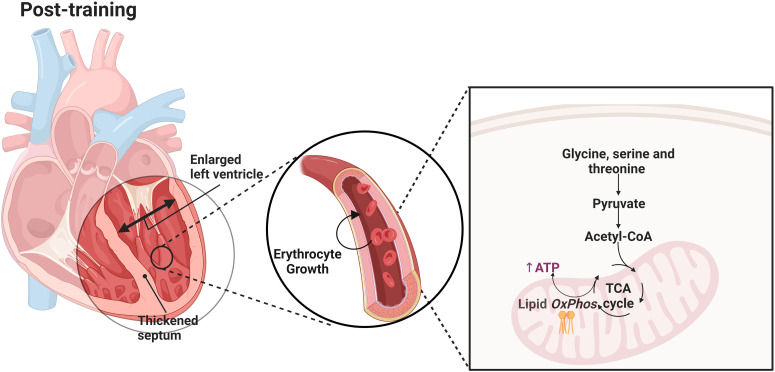
Schematic diagram of the mechanism.

During physiological cardiac remodeling, cardiomyocytes may undergo moderate hypertrophy, potentially leading to an increase in mitochondrial quantity. This increase provides additional sites for oxidative phosphorylation. More mitochondria enable more electron transport and ATP synthesis reactions, thereby enhancing the efficiency of oxidative phosphorylation. Glycine, serine and threonine metabolic pathways mediate physiological cardiac remodeling by promoting erythrocyte growth and development, regulating lipid oxidative phosphorylation, and influencing cardiac metabolic status, thereby exerting profound effects on cardiac structure and function (Fig. 10).

This study provides a comprehensive analysis of the effects of training on the cardiac structure and function of Yili horses; however, several limitations should be noted: (1) Small sample size: The relatively small number of untrained Yili horses may limit the generalizability and statistical power of the results. (2) Complexity of multi-omics data: While multi-omics analyses were performed, the interactions and network relationships among different datasets remain complex and challenging to fully elucidate. (3) Unknown long-term effects: The study primarily focuses on the effects of six months of training, leaving the long-term impacts on cardiac structure and function unclear. (4) Interaction of environmental and genetic factors: The interplay between environmental and genetic factors influencing the performance of Yili horses was not fully explored. (5) Standardization of training protocols: Although training protocols were standardized, individual responses to training may vary, potentially affecting the consistency of the results. (6) Causal relationships: The study primarily observes correlations; further experiments are needed to confirm causal relationships between gene expression changes and metabolite level variations. (7) Complexity in data interpretation: The integration of multi-omics data provides valuable insights but makes identifying specific biological processes and molecular mechanisms challenging due to data complexity.

## 5 Conclusions

This study integrated transcriptomics, metabolomics, circulating miRNAs, and lipidomics to reveal the molecular regulatory mechanisms of exercise training on performance-related morphological and functional changes in Yili horses. The study tentatively identified the glycine, serine, and threonine metabolic pathways as key players in accounting for the differences in the training groups through their concerted effects on protein synthesis. Metabolomics analysis revealed significant expression of acylcarnitines and specific triglycerides in the trained groups compared with the untrained horses. These metabolites may reflect exercise-induced regulation of fatty acid metabolism and cardiac energy utilization.

The pathway diagram (Fig 10) highlights the role of glycine, serine, and threonine metabolism in cardiac remodeling, with annotations of the significant genes, metabolites, and lipid compounds identified in this study. This research provides useful data and insights into the potential molecular mechanisms underlying exercise-mediated athletic enhancement in Yili horses and lays a foundation for future optimization of training programs and animal model studies.

## Supporting Information

S1 Text Training Plan in This Study. (DOCX)

S1 Table Yili Horse Size Data. (XLSX)

S2 Table Yili Horse 1000m Speed Race Results. (XLSX)

S3 Table qPCR for mRNA and miRNA. (XLSX)
