# Supplementary material for: Metabolomics analysis and mRNA/miRNA profiling reveal potential cardiac regulatory mechanisms in Yili racehorses under different training regimens
Source: PLoS One. 2025 Jul 14;20(7):e0322468. doi: 10.1371/journal.pone.0322468 (PMC12258599; doi:10.1371/journal.pone.0322468)
Supplement: S1 Text — (DOCX) [file pone.0322468.s001.docx]

**Supplementary Materials**

**Training Plan in This Study**

The conditioning schedule for the horses was as follows:

**Week 1:** Round pen groundwork, back adaptation exercises, mounting in the stable, and leading practice.

**Week 2:** Warm-up in the round pen and on the mechanical walker for 40 min, followed by 20 min of riding.

**Week 3:** Similar warm-up, followed by 20 min of riding and practicing on fixed tracks, including serpentine, figure-eight, Z-shaped, and circular routes.

**Week 4:** Round pen groundwork, 35 min of warm-up on the mechanical walker, and 25 min of riding. Training included slow walking (800 m), trotting (800 m), and slow walking (800 m) on the track.

**Week 5:** Trotting distance was increased to 1600 m, while the other activities remained the same as for Week 4.

**Week 6:** Round pen groundwork and mechanical walker warm-up for 30 min, followed by 30 min of riding. Training included short sprints (400 m) and trotting (800 m). For the next five days, the sprint distance was increased by 50 m each day, while the trotting distance was reduced by 50 m.

**Week 7:** Warm-up, 30 min of riding, 800 m trotting, 800 m short sprints, and 800 m slow walking.

**Week 8:** Warm-up as in Week 7, followed by 1200 m short sprints on grass.

**Week 9:** Similar warm-up and track training as Week 8, with grass sprints increased to 2100 m.

**Week 10:** Warm-up, 30 min of riding, 800 m slow walking, 800 m trotting, and gradual acceleration on grass until reaching a canter.

The training plan was designed based on heart rate zones: trotting at 50–60% HRmax, galloping at 70% HRmax, and slow walking. Riding and track training sessions were implemented accordingly. The six horses in the UN group were not subjected to training and were allowed free movement in the paddock. At the end of the training period, the performance of the horses was tested by a 1000 m race. Based on the results, the top ten performers were assigned to the EXC group (average time: 75.68 ± 1.36 s), and the lower ten performers were assigned to the CON group (average time: 78.32 ± 1.97 s).
